# Identity Development in Disorientating Times: the Experiences of Medical Students During COVID-19

**DOI:** 10.1007/s40670-022-01592-z

**Published:** 2022-08-01

**Authors:** Megan E. L. Brown, Jun Hua Lim, Jo Horsburgh, Chance Pistoll, Viral Thakerar, Arti Maini, Caroline Johnson, Laura Beaton, Claire Mahoney, Sonia Kumar

**Affiliations:** 1 Medical Education Innovation and Research Centre (MEdIC), Imperial College London, London, UK; 2 Health Professions Education Unit, Hull York Medical School, University of York, York, UK; 3 Department of General Practice, Melbourne Medical School, The University of Melbourne, Parkville, VIC Australia

**Keywords:** Professional identity, COVID-19, Medical education, Kegan

## Abstract

**Introduction:**

Professional identity development is a central aim of medical education, which has been disrupted during COVID-19. Yet, no research has qualitatively explored COVID-19’s impact across institutions or countries on medical students’ identities. Kegan proposes a cognitive model of identity development, where ‘disorientating dilemmas’ prompt student development. Given the potential of COVID-related disruption to generate disorientating dilemmas, the authors investigated the ways in which COVID-19 influenced students’ identity development.

**Methods:**

The authors conducted an international qualitative study with second year medical students from Imperial College London, and third year students from Melbourne Medical School. Six focus groups occurred 2020–2021, with three to six students per group. Authors analysed data using reflexive thematic analysis, applying Kegan’s model as a sensitising theoretical lens.

**Results:**

COVID-19 has resulted in a loss of clinical exposure, loss of professional relationships, and a shift in public perception of physicians. Loss of exposure to clinical practice removed the external validation from patients and seniors many students depended on for identity development. Students’ experiences encouraged them to assume the responsibilities of the profession and the communities they served, in the face of conflicting demands and risk. Acknowledging and actioning this responsibility facilitated identity development as a socially responsible advocate.

**Conclusions:**

Educators should consider adapting medical education to support students through Kegan’s stages of development. Measures to foster relationships between students, patients, and staff are likely necessary. Formal curricula provisions, such as spaces for reflection and opportunities for social responsibility, may aid students in resolving the conflict many have recently experienced.

**Supplementary Information:**

The online version contains supplementary material available at 10.1007/s40670-022-01592-z.

## Introduction

In March 2020, medical education worldwide was disrupted because of COVID-19. Face-to-face clinical teaching and placements were suspended for many medical students around the world [1]. Whilst some final year students graduated early [2] to assist in the pandemic, clinical exposure for junior students was suspended for longer durations of time and much teaching was moved online [3]. Several authors have speculated about the negative impact of the disruption and adaptations resulting from COVID-19 on medical students’ professional identity development [4–7]. However, little empirical, in-depth qualitative study has been reported that investigates the impact of the pandemic on students’ professional identities. Whether or not the pandemic could have facilitated student identity development in any way remains unexplored. Given that professional identity development is widely recognised as a central aim of medical education [8] and is important in fostering confidence and wellbeing [9], disruption to identity development has potentially wide-reaching impacts.

Professional identity development can be broadly defined within medical education as the way each student comes to ‘think, feel and act like a physician’ [10]. Within healthcare, identity is commonly conceptualized from one of two theoretical orientations—as either a cognitive, developmental process, or as a social, interactional phenomenon [11]. Cognitive conceptualizations of identity development, which regard identity as situated within one’s mind, are common and popular within medical education [12]. Exploration of identity from a cognitive perspective allows for in-depth investigation of individuals’ experiences [12]. Kegan’s five-stage model of cognitive identity development is a theory that focuses on how the experiences of individuals influence their mental perceptions of their professional identity. In this model, later stages (‘orders of mind’ or epistemological lenses), are associated with increased independence and social maturity [13]. Table 1 provides further detail regarding the development that occurs within each stage of this model. Kegan’s model has been previously examined within medical education, with Cruess et al. suggesting stages 2–4 of the model are particularly relevant [14, 15].

**Table 1:** Kegan’s five orders of mind or epistemological lenses

*Stage of Kegan’s model/order of mind/epistemological lens*	*Description of stage*
Stage 1: ‘The magical childhood mind’	Developmental stage of early childhood. Young children cannot hold the idea that objects in the world retain the same qualities over time. This stage is a time of magic and mystery as the world inexplicably changes from moment to moment
Stage 2: ‘The self-sovereign mind’	Individuals existing within stage 2, ‘the self-sovereign mind’, emphasize their own needs, interests, and agendas as their primary goal. To self-sovereign minds, relationships are transactional—a means to an end of having their own needs and desires met—and rules or philosophies are adhered to because of external reward or punishment, not due to true belief or moral integrity
Stage 3: ‘The socialized mind’	Most adults exist within stage 3, ‘the socialized mind’, where external sources shape one’s sense of self and understanding of the world. In this stage, focus has shifted so priority is placed on the ideas, norms and beliefs of other people and the systems around us, as opposed to one’s own needs. External validation is sought to develop one’s identity, e.g., from grades or feedback, as individuals don’t have a strong, independent sense of self. For many people, social maturity halts here
Stage 4: ‘The self-authored mind’	Other adults progress their identity to stage 4, the ‘self-authored mind’. Within this stage, one’s sense of self is more independent, and individuals can define who they are without relation to other people, their relationships, or their environment. An internal sense of direction is established, where individuals become the ‘author’ of their own life course
Stage 5: ‘The self-transforming mind’	The final stage of Kegan’s model, stage 5, that is beyond what Cruess et al. deem relevant to medical trainees, is ‘the self-transforming mind’, a higher state of identity development, where one’s sense of self is constantly created and recreated through exploration of roles and beliefs with others. Individuals within this stage are still self-authoring, but also able to work with the authority of others to question the identity they hold. This is a stage that is rarely achieved and, if achieved, likely requires a great deal of time for development

We speculate that the disruptions in medical education because of COVID-19 may have influenced the cognitive process of medical student professional identity development. Though Kegan’s model of identity is principally cognitive in nature, it acknowledges the influence of an individual’s social world and experiences on inner conceptualizations of their identity. This interplay is captured by the process of ‘socialization’, which can be defined from a functionalist theoretical orientation [16] as “the social processes that bring people in line with the status quo” [17] (p. 123). As individuals interact with the social world, stressful ‘disorientating dilemmas’ prompt students to progress between the stages of Kegan’s model. In this way, a change in medical student experiences because of COVID-19 (a ‘disorientating dilemma’) has the potential to impact student identity development and socialization, in line within Kegan’s theory.

Though the impact of COVID-19 on identity development has been speculated by several authors [6, 7, 18, 19] there has been little in-depth empirical investigation of COVID-19’s impact on student identity to date. Early data from China, which has principally focused on medical student identity development during COVID-19 through the lens of career aspirations, has yielded mixed results, with Yang et al. demonstrating little impact on career attitudes [20], and Wen et al. reporting increased interest in infectious disease and respiratory specialties [21]. In the most in-depth qualitative study to date, Findyartini et al. explored the identity development of second, third, and fifth year medical students at an Indonesian institution, analysing written reflections [22]. They highlight two changes to student socialization as a result of COVID-19: (1) A shift from students viewing themselves as young adults towards viewing themselves as medical students; and (2) Changes in the way students, role models, and patients interact as a result of online learning, a shift felt to be negative in regard to developing the relational skills physicians require [22]. Though Findyartini et al. comment on Kegan’s model within their discussion, they do not apply the model consistently to the experiences of students during COVID-19. Given the focus of the model on ‘disorientating dilemmas’, application of Kegan’s model to students’ recent experiences may cast new light on how student development can best be supported. Further, in-depth, interview-based, qualitative research with international reach is yet to be conducted on the topic of identity development during COVID-19. Given this, we investigated whether and how COVID-19 has influenced the professional identity development of medical students within two international contexts, applying Kegan’s epistemological lenses to help make sense of student experiences.

## Methods

### Research Approach

We employed an interpretivist paradigm, utilising a socio-constructivist ontology and interpretivist epistemology. Within this approach, we understand reality and knowledge to be subjective, cognitive, individual constructions influenced by social forces [23, 24]. In line with this orientation, we selected a qualitative approach. We conceptualize identity as a cognitive construct influenced by social phenomena as individuals subjectively assign meaning to their experiences. We have chosen to adopt a cognitive conceptualization of identity as we are principally interested in the way in which individuals have experienced and rationalized recent disruptions.

### Context

Institutions responded variously to COVID-19, and so it is important to consider the response of the two sites within this study, Imperial College London (ICL) and Melbourne Medical School (MMS) (Supplementary Material). It is also important to consider the difference in case rates, hospitalizations and mortality rates between the UK and Australia, as this provides context for the response of each institution and our findings (Supplementary Material).

### Participants

We recruited second year medical students from ICL and third year medical students from MMS for participation in voluntary focus groups. Email invitations to participate were circulated by administrative staff. Focus group facilitators also used snowballing as a recruitment strategy.

ICL’s medical degree is an undergraduate qualification, whilst MMS’s medical degree is a postgraduate course. Whilst ICL students are four years from qualification, MMS students are one year from qualification. We intentionally sought participants from these two distinct groups to evaluate whether there were transferrable findings regarding the impact on identity development across junior and senior medical students.

Participation within the ICL arm of the study was incentivized, with each participant offered a £20 voucher for their time. No incentives were used in the MMS arm of the study. We obtained written, informed consent from participants.

### Data Collection

We held six focus groups, of 60-min duration, each comprising 3–6 medical students, between October 2020 and April 2021. One or two facilitators conducted each focus group via video conference. Where two facilitators were present, the second facilitator took field notes. Focus groups were semi-structured, with facilitators guided by a list of prompts (Electronic Supplementary Material). Focus groups were audio recorded, transcribed verbatim, and anonymized for analysis.

Malterud et al.’s qualitative concept of ‘informational power’ was used to guide sample size [25]. As a group, it was felt that, after six focus groups with 25 students (13 from Imperial and 12 from Melbourne), findings were of appropriate depth to answer the research question of this study and generate transferrable findings [26].

#### Data Analysis

Braun and Clarke’s six-step approach to thematic analysis was used to analyse focus group data inductively [27]. All authors read and reread a selection of focus group transcripts to foster familiarity with the data. MB, CP, and JL assigned descriptive preliminary codes to MMS’s data in parallel, to deepen analysis, whilst JH and VT did similarly for ICL’s data. Following descriptive preliminary coding, both sites met together to discuss their interpretations and agree on a codebook. MB systematically applied this codebook to data from both sites. Themes were critically reviewed and revised as a group, then defined and named. MB produced a narrative report, illustrating themes using participant quotes.

Reflexivity is a core component of Braun and Clarke’s analysis [27]. Researchers involved in coding were encouraged to keep reflexive diaries, documenting assumptions regarding identity, education, and reflecting upon moments of surprise during analysis. All researchers reflected on their assumptions and experiences within group discussions.

#### Theoretical Framework

During analysis, the research team immersed themselves in identity theory, through which Kegan’s developmental model of identity was identified as relevant to the study’s inductive findings. This model offers a cognitive conceptualization of identity that aligns with our constructivist approach. The researchers applied Kegan’s five epistemological lenses as sensitising concepts to help interpret data in a ‘theory informing inductive data analysis’ approach [28]. Sensitising concepts have been previously used within thematic analysis [29–31] and originated within sociology as a way of describing how theory can inform the discussion of inductively analysed data [32].

## Results

Twenty-five medical students (13 ICL and 12 MMS) participated across 6 focus groups.

The authors identified four themes which speak to the influence of COVID-19 on students’ identities: (1) Influence of the media on student identity development; (2) Influence of curricula on student identity development; (3) Impact of COVID-19 experiences on self-definition; and (4) Tensions between responsibilities. Quotes are labelled using focus group (FG) numbers and site (ICL or MMS).

### Influence of the Media on Student Identity Development

When discussing their identity development and the impact of the pandemic, medical students described the impact of healthcare workers’ public images on how they viewed themselves as future physicians. Of concern across both sites was the predominance of the ‘hero narrative’ within the media.

*“…elevating doctors to the status of heroes doesn’t help because we’re not heroes, we’re human beings who have the same needs and who need to work…”*

*ICL, FG1*

Within the UK, but not within Australia, perhaps due to case/fatality rates, war metaphors used by the media were a source of conflict for students, leading them to question the acceptability of risk within their future professional identities.

*“…the war metaphors…all of these doctors, they’re heroic soldiers in a war zone. I didn’t sign up to work in a war zone! That’s not what any of these people’s jobs is meant to be. You know, the front lines, the battle against the pandemic. That’s not what being a doctor is meant to be about.”*

*ICL, FG3*

Despite such concerns regarding physicians’ portrayal by the media, students did note increased levels of respect and appreciation, which reinforced the responsibility physicians hold within society.

*“… at the moment there’s a big focus on the NHS [National Health Service] and NHS workers and they’re getting a lot more respect from the public, so it’s one of those roles in society which people look up to…it’s nice to have that responsibility…”*

*ICL, FG2*

### Influence of Curricula on Student Identity Development

Students also highlighted the influence of changes on formal and informal curricula. A transition to distance learning reduced student identification with the profession—there was a sense that it was impossible to become a physician through online learning alone.

*“The first lockdown, when we didn’t have placement at all, I just felt disconnected…I thought I was doing a science degree…”*

*MMS, FG1*

Additionally, there were concerns from many students regarding the ways in which the pandemic had reduced opportunities to connect with professionals and role models.

*“…because of this pandemic, that professional growth has staggered…. professional growth, they define it as building networks…that’s less possible online. These networks consist of doctors who motivate you down a certain career path…open your eyes to certain principles within medicine…maybe they could even be a referee for your future job.”*

*MMS, FG2*

External validation in the form of positive patient feedback was missed by students for whom placements had been suspended. In the quote below, a student highlights the value of this validation on return to clinical placement.

*“…one of the women who came in… she was very surprised that I was there as a medical student…she actually valued it much more, and she said, “I really value you being here, trying to help me, putting yourself at risk and not earning any money for it whatsoever.” I thought that was just reaffirming my role and the part I can do...”*

*MMS, FG2*

Though students were provided with telehealth opportunities to consult patients, which they appreciated, there were some concerns telehealth negatively impacted relationships.

*“…it’s been harder for some patients to feel connected with doctors because we’ve had to move to telehealth…that may affect as well how much people trust doctors if they haven’t been able to make that connection with a regular doctor...”*

*MMS, FG2*

Reflective sessions (such as the professional practice sessions at MMS) were noted as a valuable curricula provision which helped students to process concerns about becoming a physician.

*“I enjoy our professional practice sessions…that has probably been the single thing this year that has shaped my professional identity development the most, being able to have space to talk about what worries us about becoming doctors, and then work through that with both tutors and peers...”*

*MMS, FG2*

Where in-person teaching or placements had continued, students appreciated the ability to connect with peers and educators. Further, students’ physical presence despite widespread closures reinforced to some the status of their role.

*“The fact that we were allowed to continue…highlighted the importance [of the role] for me, that I hadn’t quite realised…”*

*ICL, FG2*

### Impact of COVID-19 Experiences on Self-Definition

Students’ experiences of COVID-19 also influenced the ways in which they conceptualized their status, values, skills, and career aspirations as future physicians.

#### Status

Initially, when clinical placements were suspended, students admitted they felt purposeless. There was a tension highlighted within UK data between medical students’ status as keyworkers (and the benefits associated with this status e.g., public transport access), and the little students felt they were contributing. Where students returned to placements or volunteered, they were concerned their presence burdened physicians.

*“…medical students are classed as key workers… there’s a bit of impostor syndrome in that, we’re not deserving of it because actually, we don’t have the knowledge or skills to really help people when we’re on these placements, and we’re almost more of a burden to the staff in hospitals… we’re getting all this praise and recognition and status, even though we’re not doing anything to help.”*

*ICL, FG1*

MMS students, given their more senior status, felt more able to contribute clinically. However, some did note that, upon return to clinical placement, their role was more observational than they had previously experienced, and so less engaging.

*“The second time around, even though there is a little bit of placement and interaction…I still feel a little bit disengaged because…it’s almost completely observational…”*

*MMS, FG1*

#### Skills and Values

For many students, COVID-19 highlighted the importance of public health knowledge, person-centeredness, and continuity of care. Further, though there was scepticism from some regarding the role of telehealth, the pandemic offered students the opportunity to develop teleconsultation skills.

*“We’ve had the opportunity to adapt to telehealth along with medical professionals like GPs …. the skills we’re learning with telehealth are invaluable for future practice.”*

*MMS, FG2*

#### Career Aspirations

Regarding career aspirations, COVID-19 confirmed for many students their desire to practice as a doctor. However, the influence on speciality aspirations was mixed, with some sharing new secondary care aspirations and some prioritizing primary care. What was relatively unifying was increased consideration of work-life balance regarding future careers. COVID-19 highlighted the implications of stress, poor working conditions, and impaired wellbeing to many students, which led to thoughts about how to reduce the risk of these in future careers. Some believed primary care to be the best option for healthy work-life balance.

*“… what the pandemic has highlighted…is that family is really important to me… it made me more seriously consider GP.”*

*ICL, FG1*

Although for most students COVID-19 highlighted facets of future careers they wished to target, for others, not experiencing placements of interest during clinical placement suspensions cast doubt on career aspirations.

*“…this year was a year that I was really looking forward to because really all of the rotations are things that I’ve had an interest in…having missed a lot of them…I’m in this position of needing to find a way to experience those…so I can really see if it’s for me or not and make a decision down the line...”*

*MMS, FG2*

### Tensions Between Responsibilities

Significant portions of the focus group discussions concerned the conflict students experienced between their responsibility to protect their own and others’ health, and the responsibility to serve during the pandemic.

When considering risk, although students did consider the risk to their own health, they were most concerned with the risk to those around them; their family, those they lived with, and their friends.

*“I think with worrying about getting Covid… I often do worry about if I get it its asymptomatic and spreading in a supermarket or giving some to other people. That’s something that [I] would worry about but not really the risk to myself.”*

*ICL, FG1*

Students experienced tensions balancing their concerns about risk with their responsibilities as medical students. These responsibilities manifested in several different ways, for example, to monitor one’s own health for symptoms of COVID-19, and to contribute positively to the health of the community.

*“I haven’t seen my partner in nearly three months because to put a rural town at risk of an outbreak is too much of a risk …it’s like the responsibility to my practice, to the community, to myself…”*

*MMS, FG1*

Further, within the UK data, where restrictions on social interaction remained from March 2020 until July 2021, students also experienced conflict between the actions of their loved ones when they breached restrictions and their responsibility to reduce COVID-19 spread. Some students were concerned they were ‘missing out’ on social events because of their role and responsibilities.

*“There were quite a few of my friends…who weren’t doing medicine, who just didn’t care too much about the lockdown restrictions, and it felt like I would, I would say… “we can’t all meet up like it’s not allowed blah blah blah”, and I would feel responsible and not go, and so it’s kind of a little bit isolating…they were still my friends…I was always very torn.”*

*ICL, FG2*

Students also experienced responsibilities that were not in direct conflict with considerations of risk but were new to them. Most notable was students’ sense of responsibility in educating the public about COVID-19—a form of advocacy. Role modelling appropriate COVID-19 behaviour, e.g., mask wearing, was also considered as fundamental to the role of a future physician.

*“For me, it’s being an exemplar of whatever the guidelines and health advice are, never going outside without a mask on, never forgetting modelling good hand hygiene when you see patients, not travelling when you’re not meant to.”*

*MMS, FG1*

## Discussion

The COVID-19 pandemic has acted as a ‘disorientating dilemma’, unprecedented in magnitude and scale [33, 34]. The challenges posed by this crisis led to disruptions in the delivery of medical education and impacted the personal lives of students and their perceptions of their role in healthcare [35, 36]. Whilst the impact of disorientating dilemmas on professional development have been noted in the past [37, 38], our data offers unique insights into the way in which internal cognitive processes and social forces may contribute to professional identity development during times of disruption. Analysing our data from a constructive-developmentalist perspective, we observed that COVID-19 facilitated complex meaning-making amongst medical students. At times, COVID-19 hampered development, but, in many cases, acted as a catalyst for student identity development. The salient factors we have identified as instigating changes were (1) Loss of relationships and external validation from patients and seniors and (2) The pandemic empowering students to develop an internal sense of direction and responsibility regarding their personal values. Within this discussion, we will explore these factors and make practical recommendations for educators and institutions interested in facilitating medical student identity development during, and beyond, times of crisis.

Socialization plays an important role in shaping students’ professional identities as they interact with the world around them and progress through Kegan’s stages [15, 39–41]. We observed that COVID-19 restrictions impeded students’ access to clinical settings, which hampered socialization within the medical profession and is likely to have stifled student progression between the self-sovereign mind, and the socialized mind. Clinical placements have traditionally contributed to identity development by exposing students to the social context of clinical work [42]. This is where students become familiar with medicine’s hidden curriculum, which includes the characteristic values and norms of the profession that are eventually embodied by socialized students [15, 43]. Regarding Kegan’s model, such values and norms learnt in practice shape one’s understanding of the world and encourage transition from a self-sovereign epistemological lens to a more mature, socialized lens [44]. Our data suggests that, although students believed that distance learning was a necessary compromise, they found the overall experience suboptimal, much like students within Findyartini et al.’s research [22]. Students keenly felt their separation from patients, role models, and mentors key factors influencing socialization [39]. Within our study, removal of students from clinical placements affected their sense of solidarity with the medical profession, culminating in feelings of isolation and helplessness that reinforced a self-sovereign view of the world. This finding is consistent with descriptive studies responding to students’ identity development needs at the beginning of the pandemic [4]. Interestingly, perhaps as our research was conducted later in the pandemic, we found that, upon return to clinical placement, students experienced a sense of personal agency directed at supporting a larger cause that encouraged a shift towards a more socialized lens. Our findings add to current literature by emphasizing that a connection to the social contexts of medicine, including to role models, is necessary to maintain socialization and promote transition from self-sovereign orientations to socialized orientations.

A particularly prominent theme within our data concerns the development of students’ identities as advocates for science and patients. Physicians describe two forms of patient advocacy: agentic advocacy (providing information and helping patients to navigate healthcare systems) and activism advocacy (using their social status to raise awareness about health inequities and influence policy) [45]. Our data suggest that COVID-19 has encouraged students to develop as agentic advocates within their local communities. Socially during times of national restrictions, students described challenges where the expectations of their loved ones existed in conflict with their nascent identities as healthcare providers. In these instances, we noted students reflected on personal values as a means of adjudicating conflicting expectations. Further, students often assumed a role educating others about COVID-19. These decisions make students accountable to the community which they serve but require a degree of self-sacrifice. Complex interactions like these signify transition from Kegan’s socialized lens, where students may succumb to peer pressure, to a self-authoring lens, where an internal sense of direction guided by values rooted in social responsibility is established [46]. Wald and Ruddy speculate that dealing with dilemmas during COVID-19, such as one’s own commitment to the medical profession, may support the development of ‘a morally resilient, humanistic’ identity [47]. Our research supports this suggestion, as development of students’ identities as agentic advocates within non-healthcare settings acted to catalyze student identity development through the stages of Kegan’s model, to more developed levels than previously reported within medical students [22].

Students’ embodiment of agentic advocacy identities was facilitated by a heightened awareness of the consequences of their actions and behaviour—their responsibilities as future physicians. Yardley et al. note that responsibility and identity exist in symbiosis with one another— [48] in this study, an acceptance of one’s responsibility to the medical profession and local communities was similarly connected to identity development. It is important to highlight students nobly accepted these responsibilities in the face of risks to their personal and loved ones’ health, and relationships with those around them. This concords with Harries et al.’s research from the USA, which reported that, during the initial peak of COVID-19, 83% of 741 clinical-stage medical students wished to accept the risk of infection and return to placement [49]. However, this same study reported that only 38% of students believed they had a moral, ethical, or professional obligation to assist during the pandemic [49], a figure lower than previous hypothetical studies concerning student willingness to volunteer [50]. We challenge Harries et al.’s finding and suggest, through our qualitative analysis, that students’ motivation to return to clinical practice is not simply fuelled by self-motivated reasons (a self-sovereign orientation), but also by the responsibility students experience towards their profession, patients, and communities.

### Implications

The implications of these findings for educators and institutions are summarised in Table 2.

**Table 2:** Recommendations for educators and institutions

*Finding*	*Recommendations for practice*
**A connection to the social contexts of medicine, including to role models, is necessary to maintain socialization and promote transition from self-sovereign orientations to socialized orientations**	Involving students in remote telehealth consultations, creating virtual events that allow students to develop professional networks, and mentor-facilitated reflective sessions may help maintain social connections Creating guidance for tutors using telehealth as a teaching tool with students as to how they can actively engage students and investing time into researching telehealth as a pedagogical tool may also be of benefit Such initiatives would promote interaction with the hidden curriculum of medicine and allow students to access the external validation they may depend upon. Formal reflective sessions should be maintained beyond the current pandemic, in order to help students navigate the disorientating dilemmas which occur within medicine
**Students’ embodiment of agentic advocacy identities was facilitated by a heightened awareness of their responsibilities as future physicians**	Institutions wishing to facilitate the symbiotic relationship between responsibility and identity beyond the setting of an international pandemic may consider encouraging students to reflect on physicians’ responsibilities to patients, communities, and society within designated reflective spaces. Examples of conflicts in responsibility, such as were experienced by some students in this study, e.g., between responsibility to one’s family, and to one’s profession, could be discussed. Examples from the COVID-19 pandemic, including examples from the media, could be used in future to illustrate such tensions as a platform for discussion As developing as an agentic advocate involves educating patients, institutions may wish to consider formal training for students regarding health education and social media use to educate the public. Beyond the context of the pandemic, roles where students are able and expected to participate in health education may encourage agentic advocacy
**Responsibility, advocacy, and identity are connected, and can develop in both pre-clinical and clinical medical students**	Given this finding, institutions may wish to consider formal service-provision roles for more junior medical students that involve a higher degree of responsibility than has been typically expected of pre-clinical students during clinical rotations Where possible and desired by students, students should be supported by their institutions to assist in service-provision during times of crisis, as this enables them to fulfil the responsibilities they experience towards their profession

### Strengths and Limitations

Whilst we have highlighted transferrable findings across both groups of students in our study, ICL and MMS students do differ—they are at different stages of their training, receive different medical qualifications, and reside in countries where the pandemic response, and severity of the pandemic, differed. These differences are, however, a strength of our study, offering a source of comparison through which we were able to identify opportunities for transition through Kegan’s stages, regardless of year level, course type, or country.

The observations and recommendations we have made are highly dependent on the contemporary conceptualization of identity in medical education, which has been criticized for centring only on Eurocentric epistemologies [51, 52]. This may reduce the transferability of our findings to non-Western contexts. Future research would do well to consider identity development across a wider variety of settings.

## Conclusion

This international study set out to investigate the impact of the disruptions caused by COVID-19 to medical education on the identity development of medical students. Though previously, authors have speculated that COVID-19 may have negatively impacted student identity, our data reveals a more complex picture. Though loss of clinical contact and relationships was felt keenly, students reported development as advocates for patients, embodying the responsibilities and values of the medical profession. Several recommendations to facilitate identity development during and beyond times of crisis have been identified. Opportunities for medical students to be actively involved in clinical practice support epistemological bridge-building between self-sovereign and socialised developmental stages. Formal curricula provisions, such as reflective sessions and service learning, may encourage transition towards a self-authored lens. Institutions should consider whether they can do more to support medical students to develop their professional identities as confident, values driven, and competent advocates for patients during and beyond this pandemic.

## Supplementary Information

Below is the link to the electronic supplementary material.

Supplementary file1 (DOCX 19 kb)

Supplementary file2 (DOCX 13 kb)
