# Supplementary material for: Identity Development in Disorientating Times: the Experiences of Medical Students During COVID-19
Source: Med Sci Educ. 2022 Aug 1;32(5):995–1004. doi: 10.1007/s40670-022-01592-z (PMC9340721; doi:10.1007/s40670-022-01592-z)
Supplement: Supplementary file 1 — Supplementary file1 (DOCX 19 kb) [file 40670_2022_1592_MOESM1_ESM.docx]

Table 1. Information regarding national Covid-19 situations and institutional responses at each site.

| *Site* | *Overview of healthcare system and postgraduate training* | *National backdrop regarding Covid-19* | *Response of institution* |
| --- | --- | --- | --- |
| **Melbourne Medical School (MMS)** | Within Australia, healthcare is largely funded through the public Medicare program. Both public and private healthcare providers deliver care. Medicare is a single-payer universal healthcare scheme, funded through a range of taxation measures.  Medical education is transitioning to graduate entry, where students complete a two- to four-year undergraduate degree prior to completing a four-year MD program. MMS is one of these newer program.    Newly qualified physicians undertake an intern year (PGY1) and, often, a resident year (PGY2) upon qualification. Some trainees choose to enter basic physician training or general practitioner training directly after their internship, whilst others choose to train as a specialist in general practice, or as a specialist consultant or hospitalist after both intern and resident years. | The Australian response to the Covid-19 pandemic is led by various state governments and the Commonwealth Government of Australia. The Victorian Department of Health lead the public heath response in Victoria where the Melbourne Medical school is based. In 2020 the medical school year started in February approximately one month after the first SARS-CoV-2 case in Victoria.^1^  There were two major peaks in SARS-CoV-2 cases in Victoria. The initial peak was largely due to infection in returned travelers. It began in March 2020 with a peak of 106 new cases daily. This prompted public health officials to declare a state of emergency and escalation of Covid restrictions to their second highest level (Stage 3) by the end of March. Stage 3 restrictions required individuals to only leave their residence for essential shopping, exercise, medical care and work or study (if not possible remotely).^2^  Some restrictions were lifted in May however they were later reinstated in the second wave which started in June. During the height of the second peak Victoria had 687 cases daily.^1^ Restrictions were reinstated and escalated to their highest-level (Stage 4). Stage 4 restrictions added an 8pm to 5am curfew, a 5km travel restriction and the closure of all non-essential services^3^ to existing stage 3 restrictions.  During restrictions, elective and routine medical care was cancelled. Consultations in general practice moved from predominantly face to face consultations to telephone or online consultations.  At the start of the pandemic in Australia there were reports about lack of PPE.  By the end of Feb 2021, 909 deaths had been reported in Australia, 820 of these within Victoria.^4^ | Within Melbourne, participants’ clinical teaching was initially interrupted without changes to the medical school curriculum.  As third year placements involved vulnerable groups (aged care, pediatrics, general practice, mental health and women’s health), MMS proactively suspended these placements even prior to stay-at-home orders, with a view to re-evaluate recommencement at a later date.  As case numbers rose, a timely return to placement became less likely, hence the MMS enacted changes to the curriculum to ensure all students graduate on time. To meet course requirements for teaching and assessment, transitions to online learning and simulated clinical contact were made.  In mid-2020, discussions with clinical site coordinators and government led to recognition of students as essential workers, allowing for return to placements alongside online teaching. This recommencement was partial and consisted of restricted clinical contact hours at no more than one clinical site at a time. Students spent most of their non-clinical contact hours on a newly developed online research course offered in lieu of a 6-month research project (usually offered in their final year).  This flexibility allowed clinical hours to be compensated in 2021 in the form of a new year-long placement in place of the traditional 4th year research project. The MMS also expanded the professional practice tutorial stream to include 3rd year students . These were intended to provide ongoing support, advice, and mentorship to students. Tutorials covered topics such as ethical practice, professional identity formation and teamwork. |
| **Imperial College London (ICL)** | Within the UK, the National Health Service (NHS) is a government-funded public healthcare system, free at the point of delivery for patients.  Medical education within the UK is offered to both undergraduate students from the age of 18 on either five- or six-year courses, or to postgraduate students following completion of a relevant first degree in four-year courses. ICL offers a six-year undergraduate course.  Newly qualified physicians undertake a national postgraduate training program which lasts for two years and is known as ‘the foundation program. During the foundation program, physicians rotate through a variety of medical, surgical, psychiatric and community specialities every 4 months. On graduation from the foundation program, physicians can choose to undertake further specialist training in a specific area of healthcare to qualify as a consultant in medicine, surgery, psychiatry, or community medicine. | A nationwide lockdown was announced on 23^rd^ March 2020,^5^ although many universities transitioned to remote and online teaching and assessment about 7-10 days earlier.  In order to concentrate efforts on treating patients with Covid-19 and minimize spread of the virus elective and routine medical care was cancelled. Consultations in general practice moved from predominantly face to face consultations to telephone or online consultations.^6^  At the beginning of the pandemic in the UK there were reports about the lack of PPE for medical professionals.^7^  Final year medical students in the UK were offered the opportunity to graduate early and commence work as interim foundation year one doctors (FiY1s) from Spring 2020, prior to students commencing their first graduate post in August 2020.  There have been two major peaks in SARS-CoV-2 cases within the UK. As previously, the first nationwide lockdown commenced in March 2020. Most lockdown restrictions were lifted on 4^th^ July 2020, with hospitality businesses permitted to open and gatherings of up to 30 people legally permissible.^8^  On 14^th^ September, in response to increasing case rates, England’s restrictions were tightened, with social gatherings limited to no more than six people. Eleven days later, a curfew was imposed on hospitality establishments. During this period, local restrictions based on case rates were introduced across England, with the government operating a ‘three tier system’.^9^ Areas with higher case rates were classified as higher tiers (tier 2 or tier 3) and subject to increased restrictions. London was initially placed in tier 2.  On the 5^th of^ November, national restrictions were re-introduced in England and the country re-entered lockdown. Restrictions eased on the 2^nd of^ December, with a more restricted version of the three-tier system re-introduced.^9^ London entered tier 3 on 16^th^ December, meaning that hospitality businesses closed apart from takeaways, indoor mixing was banned, and travel outside of and into London restricted.  A fourth tier was established for high-risk areas on the 30th of December in response to the identification of a new variant of SARS-CoV-2, placing 75% of the country (including London) in tier four, with restrictions like national lockdown rules.^9^ On the 6^th^ of January 2021, the country entered national lockdown.^9^ Lockdown restrictions began to ease gradually, in line with lowering case rates and increasing rates of vaccination, on 8^th^ March 2021.^10^  By end of February 2021 123,989 deaths had been reported.^10^ | Year 1 medical students at Imperial completed their eight-day community clinical placement as planned just before the Covid-19 pandemic reached the UK.  Once lockdown began in late March 2020, all their teaching including basic sciences was converted online. Their next clinical placement was due to be two weeks in the hospital setting in May 2020, which was converted to a remote / digital-only package. This included using a serious game for digital clinical simulations, e-learning and live online sessions. Written assessments were conducted remotely from the students’ own premises.  Medical students were given ‘key worker’ status which allowed them to travel to clinical placements and use public transport during this time.  In the new academic year starting September 2020, these students entered year 2 and resumed some non-placement teaching on site in small groups, but most teaching was remote and online. From October 2020 until February 2021, they also attended community clinical placements on a rotation system to limit footfall in the GP surgery at any one time. This meant they spent 3 half days on site and the remaining 7 half days off site for their 5-day placement in year 2. The placement consisted of remote health coaching conversations with patients, remote tutorials with a GP facilitator, and taking supervised histories in GP clinics.  In December 2020, their two-week hospital placement was converted to a digital remote placement following government advice. Students could volunteer to also attend in person for those two weeks which approximately half the cohort took up.  All students in addition were part of an “academic tutor group”, typically consisting of 12 students in their year group who met with an academic tutor individually at least once a term both individually and as a group. This was for both academic and pastoral support. |

References:

1. Wang S, Liu Y, Hu T. Examining the Change of Human Mobility Adherent to Social Restriction Policies and Its Effect on COVID-19 Cases in Australia. Int J Environ Res Public Health. 2020;17(21).

2. Victoria State Government Department of Health and Human Services. Victorian coronavirus (COVID-19) data. Updated June, 2021. Retrieved June 6, 2021. https://www.dhhs.vic.gov.au/victorian-coronavirus-covid-19-data

3. Victoria State Government Department of Health and Human Services. Premier's statement on changes to Melbourne's restrictions. Published August, 2020. Retrieved June 6, 2021. https://www.dhhs.vic.gov.au/updates/coronavirus-covid-19/premiers-statement-changes-melbournes-restrictions-2-august-2020

4. Australian Government Department of Health. Coronavirus (COVID-19) at a glance- 25 February 2021. Published February, 2021. Retrieved June 6, 2021. https://www.health.gov.au/resources/publications/coronavirus-covid-19-at-a-glance-25-february-2021

5. Johnson B. Prime Minister’s statement on coronavirus (COVID-19): 23 March 2020. Gov.uk. Published March, 2020. Retrieved June 1, 2021. https://www.gov.uk/government/speeches/pm-address-to-the-nation-on-coronavirus-23-march-2020

6. NHS Improvement. Advice on how to establish a remote ‘total triage’ model in general practice using online consultations. NHS England. Updated September, 2020. Retrieved June 1, 2021. https://www.england.nhs.uk/coronavirus/wp-content/uploads/sites/52/2020/03/C0098-total-triage-blueprint-september-2020-v3.pdf

7. Mroz G, Papoutsi C, Greenhalgh T. ‘From disaster, miracles are wrought’: a narrative analysis of UK media depictions of remote GP consulting in the COVID-19 pandemic using Burke’s pentad. Medical Humanities. 2021;28:1-10.

8. Brown J. Briefing paper 9063: Coronavirus: A history of English lockdown laws. In: House of Commons Library, 2020. p.1-15.

9. Cabinet Office. COVID-19 Response- Spring 2021 (Summary). Gov.uk. Updated February, 2021. Retrieved May, 2021. https://www.gov.uk/government/publications/covid-19-response-spring-2021/covid-19-response-spring-2021-summary

10. Gov.uk. Coronavirus deaths in the United Kingdom. Gov.uk. Updated May, 2021. Retrieved May, 2021. https://coronavirus.data.gov.uk/details/deaths
