# Supplementary material for: Identity Development in Disorientating Times: the Experiences of Medical Students During COVID-19
Source: Med Sci Educ. 2022 Aug 1;32(5):995–1004. doi: 10.1007/s40670-022-01592-z (PMC9340721; doi:10.1007/s40670-022-01592-z)
Supplement: Supplementary file 2 — Supplementary file2 (DOCX 13 kb) [file 40670_2022_1592_MOESM2_ESM.docx]

**Focus group prompts:**

Introduce focus group

1. What led you to study medicine?

2. What does being a member of the medical profession mean to you? How did you come to this understanding?

a. Sub prompt: What experiences before and during medical school have been critical in shaping your understanding of what it means to be a doctor?

3. How do you think society views doctors? Do you think the COVID-19 pandemic has changed this view? How has this influenced your own thinking about the medical profession?

4. In what ways, if any, have your experiences during COVID-19 influenced whether you feel part of the medical profession?

5. How has COVID-19 affected your sense of responsibility to yourself and others —patients, family, as a future doctor and the medical profession?

a. Sub prompt: Any other conflicts of responsibility outside of COVID-19?

6. Has the experience of the COVID-19 pandemic influenced your ideas about pursuing a career in medicine, the type of doctor you want to be or the speciality you are interested in?

a. Sub prompt: Think of a doctor you currently consider an exemplar of professionalism. What made you chose this person? How could the medical school support you in your ongoing professional development?
